# Strategies for cataract and uncorrected refractive error case finding in India: Costs and cost-effectiveness at scale

**DOI:** 10.1016/j.lansea.2022.100089

**Published:** 2022-10-10

**Authors:** Brad Wong, Kuldeep Singh, Rohit C. Khanna, Thulasiraj Ravilla, Subeesh Kuyyadiyil, Shalinder Sabherwal, Asim Sil, Kuldeep Dole, Heidi Chase, Kevin D. Frick

**Affiliations:** a Mettalytics, New South Wales, Australia; b Seva Foundation, India; c Allen Foster Community Eye Health Research Centre, Gullapalli Pratibha Rao International Centre for Advancement of Rural Eye care, L V Prasad Eye Institute, Hyderabad, India; d Lions Aravind Institute of Community Ophthalmology, Aravind Eye Care System, Madurai, Tamil Nadu, India; e Sadguru Netra Chikitsalaya, Shri Sadguru Seva Sangh Trust Chitrakoot, India; f Dr. Shroff's Charity Eye Hospital, New Delhi, India; g Vivekananda Mission Asram Netra Niramay Niketan, West Bengal, India; h Poona Blind Men's Association, HV Desai Eye Hospital, Pune, Maharashtra, India; i Seva Foundation, Berkeley, CA, USA; j Johns Hopkins Carey Business School, Baltimore, USA; k Johns Hopkins Bloomberg School of Public Health, Departments of Health Policy and Management and International Health; Johns Hopkins University, School of Medicine Department of Ophthalmology, USA

**Keywords:** Cataract case finding cost, Refractive error case finding cost, Vision center cost, Teleophthalmology cost

## Abstract

**Background:**

India has the largest number of individuals suffering from visual impairment and blindness in the world. Recent surveys indicate that demand-based factors prevent more than 80% of people from seeking appropriate eye services, suggesting the need to scale up cost-effective case finding strategies. We assessed total costs and cost-effectiveness of multiple strategies to identify and encourage people to initiate corrective eye services.

**Methods:**

Using administrative and financial data from six Indian eye health providers, we conduct a retrospective micro-costing analysis of five case finding interventions that covered 1·4 million people served at primary eye care facilities (vision centers), 330,000 children screened at school, 310,000 people screened at eye camps and 290,000 people screened via door-to-door campaigns over one year. For four interventions, we estimate total provider costs, provider costs attributable to case finding and treatment initiation for uncorrected refractive error (URE) and cataracts, and the societal cost per DALY averted. We also estimate provider costs of deploying teleophthalmology capability within vision centers. Point estimates were calculated from provided data with confidence intervals determined by varying parameters probabilistically across 10,000 Monte Carlo simulations.

**Findings:**

Case finding and treatment initiation costs are lowest for eye camps (URE: $8·0 per case, 95% CI: 3·4–14·4; cataracts: $13·7 per case, 95% CI: 5·6–27·0) and vision centers (URE: $10·8 per case, 95% CI: 8·0–14·4; cataracts: $11·9 per case, 95% CI: 8·8–15·9). Door-to-door screening is as cost-effective for identifying and encouraging surgery for cataracts albeit with large uncertainty ($11·3 per case, 95% CI: 2·2 to 56·2), and more costly for initiating spectacles for URE ($25·8 per case, 95% CI: 24·1 to 30·7). School screening has the highest case finding and treatment initiation costs for URE ($29·3 per case, 95% CI: 15·5 to 49·6) due to the lower prevalence of eye problems in school aged children. The annualized cost of operating a vision center, excluding procurement of spectacles, is estimated at $11,707 (95% CI: 8,722–15,492). Adding teleophthalmology capability increases annualized costs by $1,271 per facility (95% CI: 181 to 3,340). Compared to baseline care, eye camps have an incremental cost-effectiveness ratio (ICER) of $143 per DALY (95% CI: 93–251). Vision centers have an ICER of $262 per DALY (95% CI: 175–431) and were able to reach substantially more patients than any other strategy.

**Interpretation:**

Policy makers are expected to consider cost-effective case finding strategies when budgeting for eye health in India. Screening camps and vision centers are the most cost-effective strategies for identifying and encouraging individuals to undertake corrective eye services, with vision centers likely to be most cost-effective at greater scale. Investment in eye health continues to be very cost-effective in India.

**Funding:**

The study was funded by the Seva Foundation.

**Research in context**

**Evidence before this study**

We searched PubMed and recent systematic reviews, including the *Lancet Global Health* Commission on Global Eye Health, for articles published between 1 January 2001 and 31 December 2021 that estimated the cost and / or cost-effectiveness of strategies to identify those in need of cataract surgery or spectacles for uncorrected refractive error. We used search terms for the specific interventions analyzed in this report and general terms associated with identifying patients in need of services such as “screening”, “case finding” and “outreach”. We confined the search to English language articles and those focusing on low-and-middle income countries. The search identified a large body of literature exploring the cost-effectiveness of cataract surgery, with little attention paid to the mechanism for identifying patients. The literature on correcting refractive error largely concerns screening of children, where various intervention configurations have been tested. Cost or cost-effectiveness analysis of primary eye health centers and door-to-door screening were few and of limited geographic scope. No studies examined the costs or cost-effectiveness of teleophthalmology for cataracts or refractive error. The overall evidence base provides limited guidance on which strategies are most cost-effective for identifying patients with cataracts or uncorrected refractive error at scale in low-and-middle income countries.

**Added value of this study**

To the best of our knowledge, this is the first study to examine the costs and cost-effectiveness of multiple case finding strategies in eye health at scale. The costing data cover 1·4 million patient visits at 355 primary eye care facilities, 330,000 children screened at school, 310,000 people screened at eye camps and 290,000 people screened via door-to-door campaigns over one year across 11 different states and territories of India. This study's novel contribution is the focus on the relative costs of different strategies for case finding and treatment initiation, using data that represents wide geographical coverage, a broad scope of services and substantial scale of people screened.

**Implications of all the available evidence**

At the scale considered in this study, our results indicate that vision centers and eye camps are most cost-effective in terms of identifying and incentivizing patients to initiate corrective treatment and DALYs avoided. More research is required to ascertain the cost-effectiveness of teleophthalmology. The overall evidence base indicates that investment in eye health remains very cost-effective in the Indian context. While the results are from India, they are likely relevant to other countries in the region, such as Bangladesh, which have similar levels of visual impairment, income per capita and population density, three important drivers of costs and cost-effectiveness.

Alt-text: Unlabelled box

## Introduction

India has seen substantial reduction in the prevalence of blindness and visual impairment over the last 30 years,^1^ with much of the improvement attributable to investments in surgical output, which expanded 750% from 1981 to 2012.^2^ Today, surgical supply is no longer the main constraint. In the most recent national blindness and visual impairment survey, only 8% of those with cataracts reported lack of access, with another 8% citing denial by the provider as the reasons why they did not seek corrective eye services.^3^ The remaining 84% reported constraints that can be broadly classified as ‘demand based’ such as no felt need, lack of person to accompany and fear. Similar findings have been documented for addressing refractive error.^4^^,^^5^ The implication is that mere supply is unlikely to substantially reduce the burden of blindness and visual impairment by itself. The country also needs to scale strategies that identify and encourage individuals with visual impairment to proactively initiate treatment. Because India has the largest number of individuals suffering from blindness and visual impairment of any country,^6^ improvements in India have a non-trivial bearing on the global burden of eye disease.

There is limited evidence comparing the cost-effectiveness of various visual impairment case finding strategies in India or other low-and-middle-income countries. The literature examining the cost of cataract surgery has adopted a mostly absent or incidental focus on the mechanisms for identifying patients and incentivizing surgery.^7^, ^8^, ^9^, ^10^, ^11^, ^12^, ^13^, ^14^, ^15^ A recent Cochrane review^16^ on strategies to improve access to cataract surgery in low-and-middle-income countries identified only two high quality studies,^17^^,^^18^ neither of which conducted cost-effectiveness analyses. The literature on refractive error primarily concerns strategies targeted at children such as school eye screening and variants.^15^^,^^19^, ^20^, ^21^, ^22^, ^23^, ^24^, ^25^, ^26^ Consistent with a recent systematic review,^27^ we identified only one economic analysis of teleophthalmology in a low-and-middle-income country.^28^ This study concerned the use of telemedicine for diagnosing diabetic retinopathy. Only a handful of studies have examined the costs of other case finding strategies such as health facilities^29^^,^^30^ or community outreach.^31^^,^^32^ In these instances, the geographical scope has been limited to a particular facility, hospital or locality with uncertainty about how these might generalize at scale.

**Figure 1:**
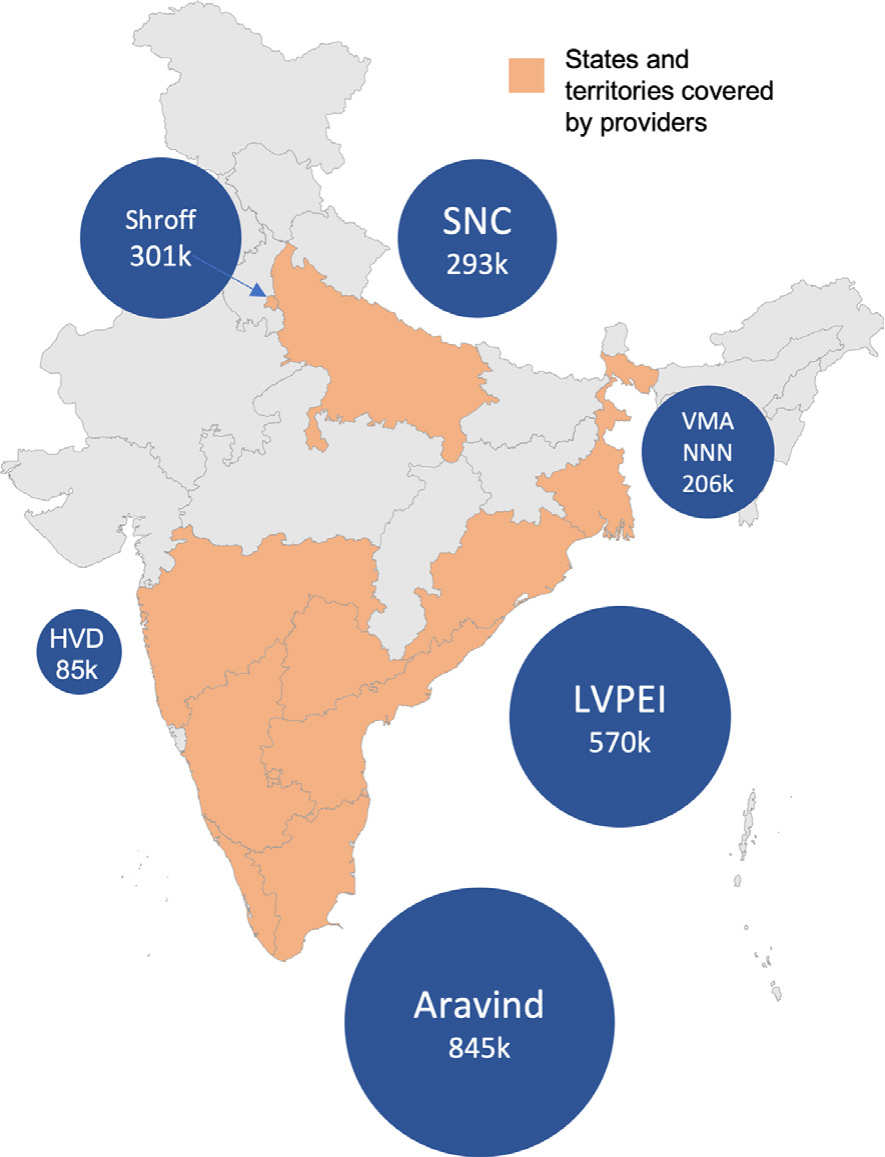
States and territories where providers have some operations. Size of bubble equals number of people covered by costing data in this study. Providers are Aravind Eye Care System (Aravind), H.V. Desai Eye Hospital (HVD), LV Prasad Eye Institute (LVPEI), Sadguru Netra Chikitsalaya (SNC), Dr. Shroff's Charity Eye Hospital SCEH) and Vivekananda Mission Asram Netra Niramay Niketan (VMANNN).

This study aims to address this gap by conducting comparable comprehensive micro-costing analyses, from the provider perspective, of five different strategies for case finding. The assessed interventions are primary eye health facilities (also known as vision centers), school screening, door-to-door eye screening, eye camps and teleophthalmology within vision centers (see Supplementary Materials, S1 for detailed description of interventions including activities, patient flows and descriptive statistics) Using administrative and financial records, augmented with semi-structured interviews, we compile data from six of the largest eye health providers in India. These providers delivered services in eleven Indian States and territories and screened at least 2·3 million people over one year (Figure 1). The costing data covers 1·4 million people seen in 355 vision centers, 330,000 children screened at school, 310,000 people screened at eye camps and 290,000 people screened via door-to-door campaigns. The providers dispensed 330,000 spectacles and performed 142,000 cataract surgeries due to these interventions.

The data enable us to report the costs of five interventions, and the cost per disability-adjusted life year (DALY) averted of four. We also report *case finding and treatment initiation costs*, which we define as all costs up until the point the patient initiates corrective eye services, either spectacles for uncorrected refractive error (URE) or surgery for cataracts. Conceptually, this includes engaging the population at risk, diagnosing those with visual impairment and converting diagnosis to treatment. This cost has analogues in other health domains such as tuberculosis^33^ but is under-considered in eye health. Since access is no longer the main constraint, one of the most important drivers of cost-effectiveness in India is the difference in the cost of case finding and treatment initiation. Scaling the most cost-effective strategies will be key to reducing the prevalence of blindness and visual impairment in India, the region, and the world.

## Methods

### Costing

Cost and administrative data were provided by six eye health care providers in India - Aravind Eye Care System (Aravind), H.V. Desai Eye Hospital (HVD), LV Prasad Eye Institute (LVPEI), Sadguru Netra Chikitsalaya (SNC), Dr. Shroff's Charity Eye Hospital (SCEH) and Vivekananda Mission Asram Netra Niramay Niketan (VMANNN). The six eye healthcare providers collectively operated 355 vision centers which saw 1,373,925 people in the financial year 1 April 2019 to 31 March 2020. The number of people screened via eye camps, school screening and door-to-door campaigns was approximately 300,000 per strategy (see Supplementary Materials S1). Across all strategies and providers, 478,276 people were diagnosed with URE of which 330,360 were provided with spectacles. There were 223,093 individuals diagnosed with cataract, of which 141,962 led to surgery.

Our aim was to collect the full financial costs of each strategy. Cost data for the five interventions were collected over the period June to September 2021 via a detailed spreadsheet template. This process was supplemented by a series of semi-structured interviews to ensure consistency and data quality (Supplementary Materials S2). Providers were instructed to provide data for all relevant activities in the financial year 2019–2020. This was done for ease of reporting, and to ensure results were not materially impacted by the COVID-19 pandemic and associated policy responses. The only exception to this was door-to-door screening, which was typically started by providers during 2020 and 2021, partially due to the pandemic. In this case, providers were asked for reliable cost and outcome data for a period of their choosing. Two providers had multi-year projects of school screening running until 2019, and the data were adjusted to reflect one year of activity. Costs were provided in 2020 INR and converted to 2020 USD at a market exchange rate of 70·42 INR to USD.^34^

Data were standardized to 8–16 different cost categories. For all interventions except teleophthalmology we report costs per 100,000 people screened. For vision centers and teleophthalmology we report costs per facility since this is likely to be a useful unit of analysis for planners of eye health networks. Point estimates were calculated from pooled data, with confidence intervals estimated by varying each cost probabilistically across 10,000 Monte Carlo simulations (Supplementary Materials S3). For reporting purposes in the main text, all costs are categorized into one of four categories: i) planning and preparation ii) operational staff iii) clinical equipment and consumables iv) operating expenses (e.g. travel, incidentals, rent, utilities). To ensure institutional privacy, we committed to reporting only pooled costs for each strategy.

To determine case finding and treatment initiation costs requires rules for apportionment of costs. In this study, all costs, with some exceptions, were apportioned to one of three categories based on the intervention-specific share of total *diagnoses*: URE, cataracts and other. For vision centers, any equipment primarily used for observing the optic nerve or retina (such as a fundus camera) was apportioned wholly to ‘other’. For eye camps and door-to-door screening, provider subsidized patient travel costs specifically for cataract surgery were apportioned wholly to cataracts.

In the final step, costs for URE were divided by the number of spectacles provided to estimate the URE case finding and treatment initiation cost. Similarly, the total costs of cataract were divided by the number of surgeries done to estimate the cataract case finding and treatment initiation cost. For school screening we only focus on URE requiring spectacles given the very low prevalence of cataracts in school age children. Case finding and treatment initiation costs for other eye health conditions are not reported since they represent a mix of diverse eye ailments that do not necessarily generalize and only represent 9% of total eye diagnoses across providers. This step ensures all costs are apportioned to an outcome (spectacles, surgery or other) including diagnosis costs of individuals not initiating the appropriate corrective services. Case finding and treatment initiation costs include preparation, screening, diagnosis, and referral follow-up but not the costs of the surgery or spectacles. By construction, it embeds the costs to diagnose those with refractive error or cataracts but who do not initiate the appropriate corrective intervention. We are unable to capture the small proportion of individuals who decide to initiate treatment at a later stage, and therefore our results likely represent an upper bound value of resources required for diagnosis leading to treatment. Formulas for calculating case finding and treatment initiation costs are presented in Supplementary Materials S4.

### Cost-effectiveness analysis

We conduct a cost-effective analysis estimating incremental societal cost per DALY averted. We include four case finding interventions (all except teleophthalmology) plus an assumed standard of care of ‘no intervention’ i.e. assuming that in the absence of the case finding strategies, URE or cataracts would otherwise not be treated. We assess this is the most appropriate counterfactual since it is unlikely patients identified by these case finding strategies would have been otherwise served in the public health system. Public primary eye health services have been historically underfunded in India. During the 2000s and 2010s, India failed to meet targets to establish 4000 and 5000 vision centers in the country's 10^th^ and 11^th^ Five Year plans respectively.^35^ While data are scarce, it is likely that India has approximately only 10% of the required 26,400 vision centers^36^ to serve the entire population. Even at these low coverage levels, NGOs, including the providers in this study, deliver the majority of eye health services in the country.^37^^,^^38^

Moreover, it is unlikely that the patients would have self-presented at the providers’ hospitals for treatment. The broad *modus operandi* of the providers has been to target unserved rural populations.^39^ For example, as SNC scaled their outreach operations between 2002 and 2013, they were able to increase cataract surgeries by 460% without any decrease in those self-presenting at the main hospitals.^2^ Furthermore, demand based factors are the major barriers to seeking appropriate eye care.^3^, ^4^, ^5^ Without the subsidized transport and counselling delivered by providers it seems unlikely that patients would self-present at distant hospitals. We test the importance of this assumption in sensitivity analyses (Supplementary Materials S6).

The cost-effectiveness analysis takes the perspective of a social planner who is aiming to maximize health outcomes from a fixed budget, similar to the framing used in identifying sectoral best buys^40^ or best buys across all health sectors.^41^ While each strategy targets different populations with varying prevalence of eye disease, we compare all strategies in the cost-effectiveness analysis because the reality of opportunity costs means that devoting resources to one type of strategy leaves fewer resources for alternatives. Moreover, the limited cost-effectiveness evidence on case finding strategies suggests it is important to identify, at a broad level, which intervention incrementally averts DALYs at lowest societal cost.

Using the results from the costing analysis plus other parameters drawn from literature or government documents, we calculate societal cost per DALY point estimates deterministically and confidence intervals using probabilistic analysis. Costs are the sum of case finding and treatment initiation costs, the costs of spectacles or cataract surgery and patient level costs^14^^,^^42^ where relevant. Each case of URE addressed with spectacles is assumed to avert 0.02 DALYs, and each case of cataract addressed, 0.85 DALYs. These figures are calculated from Global Burden of Disease disability weights^43^ and each condition's distribution of visual acuity as presented in the National Blindness and Visual Impairment Survey.^3^ The longevity of benefits for addressing URE is 3 years, the estimated useful life of a pair of glasses. The longevity of benefits for addressing cataract is 19.9 years the average life expectancy of those suffering from cataracts in India.^44^ DALYs are discounted at a rate of 3% following recommendations provided by Wilkinson and colleagues.^45^ All other parameters used in the cost-effectiveness analysis are noted in Supplementary Materials S6.

One-way sensitivity analyses are conducted by varying parameters related to discount rates, costs and the counterfactual case finding rate. Using results from the probabilistic simulation, cost effectiveness acceptability curves are calculated via the methodology presented in Fenwick and colleagues.^46^ Since school screening and door-to-door are weakly dominated, we include only eye camps (incremental to baseline) and vision centers (incremental to eye camps).

### Role of the funding source

The funder of the study had no role in study design, data analysis or data interpretation. One co-author employed by the funder (KS), was present during data collection interviews to facilitate conversation, and reviewed the draft manuscript. Another employee of the funder (HC) reviewed the final manuscript. The lead author had full access to all the data in the study and had final responsibility for the decision to submit for publication.

## Results

### Costing: Eye camps and vision centers have the lowest case finding and treatment initiation costs

Table 1 presents costing results for four interventions. School screening has the lowest cost, requiring only $86,000 per 100,000 screened. Vision centers and door-to-door require approximately $300,000 per 100,000 screened, while eye camps are the costliest at $386,000 per 100,000 screened.

**Table 1:** Annualized costs per 100,000 screened, vision centers, eye camps, door-to-door, school screening.

Category	Vision Centers	Eye Camps	Door-to-Door	School Screening	Description
Planning and preparation ($ per 100,000 screened)	25,049	119,154	14,212	3,800	Planning for screening activities, including engaging with community or schools, promotion, identifying and renovating buildings for vision centers.
Clinical equipment ($ per 100,000 screened)	56,947	18,585	4,227	12,599	Clinical equipment, tools and consumables used during screening.
Human resources ($ per 100,000 screened)	143,190	100,594	32,906	55,010	Salaries of staff required in screening.
Other operating expenses ($ per 100,000 screened)	77,299	147,408	45,586	14,853	Rent, utilities and equipment maintenance for vision centers. Staff travel, incidentals, refreshments, patient travel for other strategies. For eye camps and door-to-door much of the cost is for patient travel for cataract follow up and surgery.
Vision Center follow up for refractive error	0	0	202,816	0	For door-to-door screening only
**Total case finding and treatment initiation costs ($ per 100,000 screened)**	302,485 (95% CI:225,355-400,289)	385,742 (95% CI: 170,111-699,504)	299,748 (95% CI: 243,637-511,093)	86,262 (95% CI: 45,555-146,067)	
Number of people screened	1,373,925	307,718	286,652	332,237	
Number of spectacles provided per 100,000 screened	17,574	17,329	9,563	2,459	
Number of cataract surgeries performed per 100,000 screened	5,409	17,752	4,543	na	
Cost per person screened ($)	3·0 (95% CI: 2·3-4·0)	3·9 (95% CI: 1·7-7·0)	3·0 (95% CI: 2·4-5·1)	0·9 (95% CI: 0·5-1·5)	
Case finding and treatment initiation cost: URE ($)	10·8 (95% CI: 8·0-14·4)	8·0 (95% CI: 3·4-14·4)	25·8 (95% CI: 24·1-30·7)	29·3 (95% CI: 15·5-49·6)	
Case finding and treatment initiation cost: cataract ($)	11·9 (95% CI: 8·8-15·9)	13·7 (95% CI: 5·6-27·0)	11·3 (95% CI: 2·2 to 56·2)	na	

Notes. Costs are reported as annualized values in 2020 USD using a 3% discount rate. All data were provided by six Indian eye health providers for the Indian financial year 2019-20, except for door-to-door screening where data is from most recent year available. URE = uncorrected refractive error. Case finding and treatment initiation cost is defined as all costs required to identify and incentivize those with eye ailments to take corrective action, spectacles for URE and surgery for cataracts (see Supplementary materials S4 for detailed formula and step-by-step calculations).

Cost profiles vary substantially across strategies reflecting underlying differences in their operating models. Vision centers have the largest human resource ($143,000 per 100,000 screened) and equipment requirements ($57,000 per 100,000 screened) of any strategy. This reflects their position as a permanent offering of quality primary eye health services. For eye camps, the largest cost category is ‘other operating expenses’ estimated at $147,000 per 100,000 screened. This mostly reflects substantial patient subsidized travel costs for cataract surgery. Planning and preparation costs are also large, estimated at $120,000 per 100,000 screened, due to the substantial promotion activities conducted to encourage attendance at the camps. For door-to-door, the largest costs relate to follow up at the vision centers for those diagnosed with refractive error. For school eye screening the largest cost category is human resources, approximately $55,000 per 100,000 screened.

The case finding and treatment initiation cost summarizes how cost-effective each strategy is at converting these activities to meaningful clinical outcomes. The results indicate that case finding and treatment initiation costs are lowest for eye camps (URE: $8·0 per case, 95% CI: 3·4–14·4; cataracts: $13·7 per case, 95% CI: 5·6–27·0) and vision centers (URE: $10·8 per case, 95% CI: 8·0–14·4; cataracts: $11·9 per case, 95% CI: 8·8–15·9). Door-to-door screening has a similar case finding and treatment initiation cost for cataracts at $10·7 per case albeit with a wide confidence interval (2·3–55·6). Case finding and treatment initiation costs for URE are higher for door-to-door ($25·8 per case, 95% CI: 24·1 to 30·7) and school screening ($29·7 per case, 95% CI: 15·9 to 49·8).

### Costing: Vision centers cost $11,700 to operate per year; adding teleophthalmology capability adds a further $1,270 to operating costs

We report costs at the facility level for vision centers and teleophthalmology (Table 2). The results indicate that the average annualized cost of case finding and treatment initiation for a vision center is $11,707 (95% CI: 8,722–15,492), with human resources making up 47% of the cost base, and other expenses, such as rent and utilities, comprising approximately 26% of total costs. Spectacles are procured and then sold at vision centers and so for completeness we report the cost per vision center including procurement of spectacles at $14,954 (95% CI: 11,782 to 18,913).

**Table 2:** Annualized costs of vision centers and incremental teleophthalmology costs, per facility.

Category	Estimate	Description
Panel A: Vision Centers		
Planning and preparation ($ per facility)	969	Planning, site identification, promotion, activities to prepare space such as renovations, furniture, fittings, personnel hiring costs and training.
Clinical equipment ($ per facility)	2,204	Equipment and consumables required to perform clinical tasks including IT.
Human resources ($ per facility)	5,542	Salaries of staff managing and providing services.
Other operating expenses ($ per facility)	2,992	Rent, utilities, internet, cleaning, travel, maintenance.
**Total vision center costs for case finding and treatment initiation ($ per facility)**	**11,707 (95% CI: 8,722–15,492)**	Excludes spectacles and surgeries.
**Total vision center costs for case finding and treatment initiation plus procurement cost of spectacles ($ per facility)**	**14,954 (95% CI: 11,782–18,913)**	
Panel B: Teleophthalmology		
Planning and training ($ per facility)	57	Planning for establishing teleophthalmology including training.
Additional IT equipment and software ($ per facility)	164	Additional hardware and software that would be required to enable teleophthalmology such as a camera, patient electronic medical record software.
Doctor salary ($ per facility)	766	The average salary for doctors who consult remotely.
Additional internet costs ($ per facility)	284	Vision centers with teleophthalmology often require dedicated fixed line broadband. This category reflects these additional charges.
**Incremental Costs of Teleophthalmology ($)**	**1,271 (95% CI: 181 to 3,340)**	

Notes. Costs are reported as annualized values in 2020 USD using a 3% discount rate. All data were provided by six Indian eye health providers for the Indian financial year 2019–20. See Supplementary Materials S5 for detailed methodology on teleophthalmology costing.

Adding teleophthalmology capability to a vision center is estimated to cost an additional $1,271 (95% CI: $181 to 3,340) per year. More than 80% of the incremental costs are for internet charges and additional doctor time, 1–3 minutes per patient screened on average. All providers noted that additional doctor time was required to establish teleophthalmology. However, providers differed on which costs were incremental for teleophthalmology with respect to planning, software, hardware and internet with some indicating that for operational reasons they would incur the same expenses whether they adopted teleophthalmology or not.

Since teleophthalmology was always implemented in conjunction with a vision center, outcome data could not be reliably attributed to teleophthalmology alone. We conduct a supplementary scenario analysis to determine how effective this intervention would need to be to deliver case finding and treatment initiation costs similar to other interventions (Supplementary Materials S5). The results indicate that teleophthalmology would need to lead to 54 to 95 more spectacles being provided per facility per year (16–27% of the unfulfilled prescriptions) and 26 to 47 more cataract surgeries (17–29% of cataract surgeries recommended but not taken up) to be equally as cost-effective in case finding and treatment initiation as the most efficient alternative strategies.

### Cost-effectiveness analysis: Eye camps and vision centers are cost-effective at averting DALYs

Results of the cost-effectiveness analysis are presented in Table 3. Both school screening and door-to-door are weakly dominated, leaving only eye camps and vision centers on the efficient frontier. The results indicate that camps have ICER of $143 per DALY (95% CI: 93–251) compared to baseline. Compared to eye camps, vision centers generate an incremental cost of $5,467,344 and 20,835 incremental DALYs avoided for an ICER of $262 (95% CI: 175–431). Since case finding and treatment initiation costs for URE and cataracts are similar between vision centers and camps, the main driver of the difference in cost-effectiveness is the fact that camps identify and initiate a substantially larger proportion of people with cataracts to undertake surgery. Since surgery has superior cost-effectiveness than providing spectacles, overall cost per DALY is lower for camps. Nevertheless, ICERs for both interventions are substantially lower than the 1xGDP per capita threshold often used for assessing cost-effectiveness.

**Table 3:** Cost-effectiveness analysis of case finding strategies.

	Total Costs (USD)	Total DALYs averted	Incremental Costs (USD)	Incremental DALYS averted	ICER (USD per DALY)
Baseline, standard care	0	0	na	na	na
School Screening	278,303	178	Weakly dominated		
Door-to-Door	2,304,941	11,669	Weakly dominated		
Eye Camps	6,826,524	47,599	6,826,524	47,599	143 (95% CI: 93-251)
Vision Centers	12,293,868	68,435	5,467,344	20,835	262 (95% CI: 175-431)

Notes. Costs represent societal costs of operating each intervention from 1 April 2019 to 30 March 2020. Costs are the sum of case finding and treatment initiation costs, costs of spectacles and cataract surgery and, patient costs to access treatment. DALYs averted are estimated from administrative data on number of spectacles provided and cataract surgeries performed. DALYs and costs are discounted at a rate of 3% (Supplementary Material S6). Confidence intervals estimated by varying parameters across 10,000 Monte Carlo simulations (Supplementary Material S3 and S6).

One-way sensitivity analyses (Supplementary Materials S7) indicate that ICERs for eye camps vary from $107 to $221 per DALY averted across scenarios. Vision center ICERs range from $204 to $369 per DALY averted. In all scenarios, school screening and door-to-door are dominated or weakly dominated. ICERs are most sensitive to the choice of discount rate. Changes to the standard of care assumption increase ICERs for eye camps relative to baseline, with a cost per DALY averted of $150, $165 and $221 assuming 10%, 20% and 30% respectively of URE and cataract patients self-present at hospitals. Overall, results are robust to different assumptions of costs, baseline case finding rates and discount rates.

**Figure 2:**
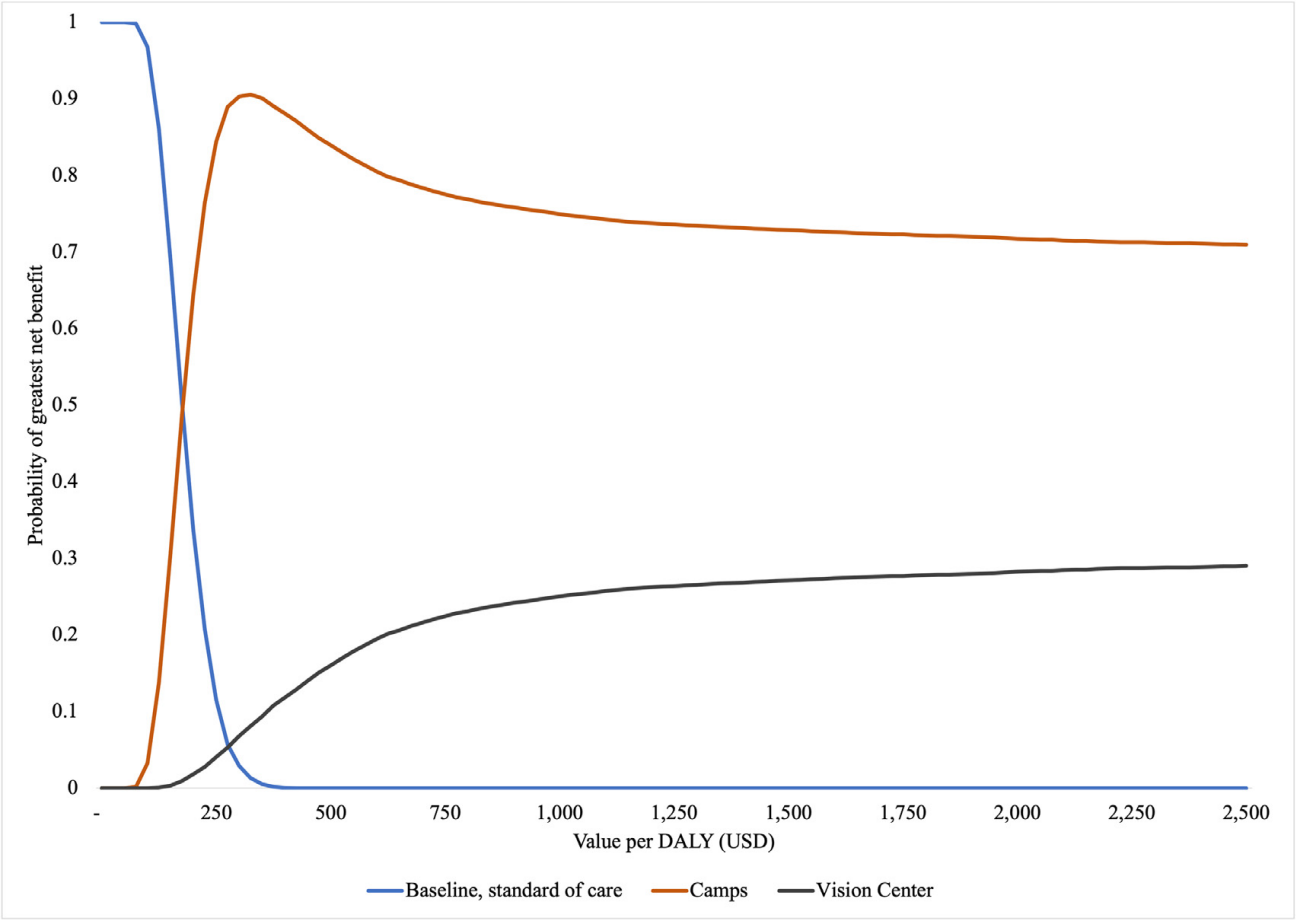
Cost-effectiveness acceptability curves comparing baseline, eye camps and vision centers. The figure depicts the probability of each intervention having the greatest incremental net benefits at various DALY valuations. Eye camp costs and DALYs are incremental to the baseline, standard of case. Vision center costs and DALYs are incremental to eye camps.

Probabilistic sensitivity analysis, as represented in the cost-effectiveness acceptability curves (Figure 2), confirms the point estimates and one way sensitivity results. Continuing the standard of care has the highest probability of having the largest incremental net benefits only at very low DALY valuations (< $175). At these low levels, where benefits are assigned little value, baseline care is superior primarily because it has the lowest cost. Beyond this threshold, eye camps always have the highest probability of the largest incremental net benefits with the maximum probability, 90%, at a DALY valuation of $350. The likelihood of vision centers having the largest incremental net benefits increases as DALY valuations rise. However, in the range considered (up to $2,500 per DALY) the probability never exceeds 30%.

## Discussion

Using data provided by six eye health care providers in India, this paper estimates the costs of five interventions implemented at scale to identify those with URE or cataracts and encourage them to take appropriate action. The data cover substantial scale and a wide geography – 2·3 million people screened across 11 states and union territories of India.

The results indicate that eye camps, vision centers and door-to-door screening are similarly cost-effective at identifying those with cataracts and incentivizing them to surgery. The former two are also the most cost-effective for identifying uncorrected refractive error and encouraging the uptake of spectacles. In contrast, URE case finding and treatment initiation costs for door-to-door and school screening are up to four times as costly as competing strategies. These higher costs are explained by the fact that in door-to-door screening URE are on-referred to a vision center which requires further costs. For school screening, costs are high due to the lower prevalence of URE in school children compared to the general population.

Case finding mechanisms for each strategy influence the outcomes and relative share of costs, and the costs per 100,000 screened. Vision centers have large human resource requirements, and incur costs that other strategies do not, such as rent and utilities. Additionally, vision centers have the largest equipment costs of all strategies, reflecting their ability to deliver more complex clinical functions. Annualized planning and preparation costs are modest because vision centers are long-lasting with upfront costs amortized over more than a decade. Due to the ability to attract a large number of patients, case finding and treatment initiation costs are among the lowest of all strategies.

Eye camps target those who suspect they have eye problems. This requires substantial resources dedicated to planning, notably promotion, to make sure communities are aware of the camp when it is operating. Moreover, because of the nature of the target group, who self-select to attend the screening, eye camps convert the largest number of people screened to spectacles or surgery. This leads to a substantial share of subsidized patient travel for cataracts. Overall costs are highest, but high rates of conversion means that the case finding and treatment initiation costs are comparable to door-to-door and vision centers.

In contrast, door-to-door is relatively low cost to implement *at the initial screening stage* with moderate planning, minimal clinical equipment and human resources required, reflecting its agile and simple operating model. Without follow up costs, the costs per person screened are only $1.0, less than a third as costly as camps. However, follow up costs to confirm diagnoses for those with refractive error are substantial, leading to large case finding and treatment initiation costs for URE. Nevertheless, case finding and treatment initiation costs for cataracts are low, suggesting that the main value of door-to-door lies in cost-effectively identifying those with cataracts and encouraging them to have surgery.

School screening generates the lowest cost per person screened due to the natural congregation of hundreds of children in one location. However, school aged children have fewer eye problems than the general population, meaning that case finding and treatment initiation costs for spectacles are effectively 3-4x as large for this intervention as eye camps and vision centers.

The comparison between strategies provides some potential operational improvements that would increase efficiency. For example, the high costs of URE case finding in door-to-door screening is primarily due to the need for patients to visit vision centers for additional diagnostics. In contrast, those diagnosed with URE in vision camps are typically provided spectacles without the need for further consultation leading to case finding and treatment initiation costs that are a quarter as large. Therefore, one potential cost saving initiative would be for door-to-door health workers to carry ready-made glasses that can be distributed to some patients to avoid further referral costs. A school screening intervention in India was able to lower costs by up to 15% due to the provision of ready-to-use spectacles.^26^ Providing ready-to-use spectacles would need to be balanced against any potential deterioration in clinical outcomes.

This report noted an average annual cost of a vision center of $11,707 per year, excluding spectacles and $14,954 with spectacles. Adding teleophthalmology capability increases annual costs by $1,271 per year. While these embed some differences in service provision between organizations, these cost estimates provide the first benchmark of an average vision center across multiple states and territories of India, a useful starting point for budgeting any scale up of facilities around the country. Applying our results suggests that India should budget $230–$410 million annually (with an extra 10% for teleophthalmology), to establish and operate the estimated 26,400 vision centers^36^ required to provide adequate primary eye health services for the entire Indian population. Assuming a 100% mark up on procurement costs, net revenue from sales of glasses could cover $60–$120 million of the required funding envelope, leaving $160–$280 million to be provided by government and donors. While this is a non-trivial amount, it is at least an order of magnitude less than the estimated $7·2–$16·4 billion annual welfare and productivity losses associated with visual impairment in India.^44^

Cost-effectiveness analyses indicate that both camps and vision centers are cost-effective at averting DALYs on average. Since all strategies operate for potentially different target groups, optimal resource allocation would likely involve some mix of these strategies with the exact proportions for a given location dependant on local factors, such as remoteness, population density and proximity to surgical capacity. Importantly, vision centers experience substantially larger scale, with four times as many people seen in facilities across India than in any of the remaining interventions. It is uncertain if average costs and cost-effectiveness would remain comparable if providers attempted to scale coverage of other strategies to the level of vision centers, especially if interventions would need to reach increasingly remote areas to generate similar screening volumes. An exploratory analysis of the impacts of scale on average costs suggests that case finding and treatment initiation costs do rise with the number of people screened based on pooled data of the other strategies, while remaining relatively constant for vision centers. However, we caution against drawing definitive conclusions due to small sample sizes (Supplementary Material S7).

While one strength of this study is the scale and scope of providers' activities, it is also potentially a limitation. The results combine different approaches in intervention implementation, for example the modality of training in vision centers, the extent of teacher involvement in school eye screening or the use of technology. Lumping these together may obscure meaningful differences in approaches that could have implications for costs. Moreover, each provider differed in their mix of interventions, potentially reflecting providers’ inherent strengths in executing preferred strategies. Pooling data may therefore generate over representation of more cost-effective variants. To assess the extent of this overweighting, we compare headline results by intervention to costs averaged at the provider level and find that pooled costs are not systematically lower than average costs by provider, suggesting this is of limited concern.

Another limitation is that this analysis only focuses on cataracts and refractive error and did not consider other eye problems such as glaucoma or diabetic retinopathy. The data, however, indicate that URE and cataracts represent 91% of all diagnoses considered in this paper. Moreover, the data represent the year before the COVID-19 pandemic, and it is unclear how costs might change with COVID prevention protocols. Lastly, our analysis ascribes equal weights to equivalent outcomes (i.e DALYs averted) for adults and children. Emerging evidence indicates that individuals in low-and-middle-income countries may value health outcomes for children higher than equivalent outcomes for adults,^47^ which could affect the relative welfare efficiency of school screening compared to the other interventions.

The primary policy implication of this analysis is that it is likely cost-effective to continue scaling up camps and vision centers. In all cases, policy makers should be aware of the potential diseconomies as target populations become more remote. Future research could examine the relationship between scale and cost, as well as how to cost-effectively combine available strategies. Another avenue of future research would be to rigorously test the impacts of teleophthalmology on case finding and clinical outcomes. Overall, this study points to the need for careful attention to case finding strategies and their costs, particularly now that most individuals who need corrective eye services are not constrained by access.

## Contributors

BW conceptualized the study, collected and analyzed data, conducted the analysis and wrote the original draft. KS conceptualized the study, validated underlying data, conducted program administration, and reviewed drafts. HC conceptualized the study and reviewed drafts. KD, RK, SK, TR, SS, AS supervised data collection within their respective institutions and reviewed drafts. KF provided supervisory guidance and reviewed drafts.

## Data Sharing Statement

A request for data can be made to the corresponding author, citing data fields required and intended use of data. The corresponding author will forward the request to the relevant provider(s). It will be up to the discretion of the provider(s) what operational and cost data will be made available.

## Editor note

The Lancet Group takes a neutral position with respect to territorial claims in published maps and institutional affiliations.

## Declaration of interests

BW declares funding from the Seva Foundation. RK declares funding from Lions Club International SightFirst Research Grant (SFP2170/UND) and United States Agency for International Development (USAID) (Grant No CBP 033). SS declares funding from USAID (Grant No CBP 020) and Peek Vision Foundation (UK).

## Appendix Supplementary materials

Image, application 1
