# Supplementary material for: Strategies for cataract and uncorrected refractive error case finding in India: Costs and cost-effectiveness at scale
Source: Lancet Reg Health Southeast Asia. 2022 Oct 10;7:100089. doi: 10.1016/j.lansea.2022.100089 (PMC10305965; doi:10.1016/j.lansea.2022.100089)
Supplement: Supplementary file 1 [file mmc1.pdf]

Supplementary Materials for Strategies for Cataract and Uncorrected Refractive Error Case Finding in India: Costs and Cost-Effectiveness at Scale

Brad Wong, Ph.D, Mettalytics, New South Wales, Australia
Kuldeep Singh MBA, Seva Foundation, India
Rohit C. Khanna, M.D. MPH, Allen Foster Community Eye Health Research Centre, Gullapalli Pratibha Rao International Centre for Advancement of Rural Eye care (GPR ICARE), L V Prasad Eye Institute, Hyderabad, India
Thulasiraj Ravilla, MBA, Lions Aravind Institute of Community Ophthalmology, Aravind Eye Care System, Madurai, Tamil Nadu, India
Subeesh Kuyyadiyil, M.Sc PH, Sadguru Netra Chikitsalaya, Shri Sadguru Seva Sangh Trust Chitrakoot, India
Shalinder Sabherwal M.S, Dr. Shroff's Charity Eye Hospital, New Delhi, India
Asim Sil, MBBS, DNB, M.Sc, Vivekananda Mission Asram Netra Niramay Niketan, West Bengal, India
Kuldeep Dole, M.S., Poona Blind Men's Association, HV Desai Eye Hospital, Pune, Maharashtra, India
Heidi Chase, MA, Seva Foundation, Berkeley, CA, USA
Kevin D. Frick, Ph.D, Johns Hopkins Carey Business School, Baltimore, USA; Johns Hopkins Bloomberg School of Public Health, Departments of Health Policy and Management and International Health; Johns Hopkins University, School of Medicine Department of Ophthalmology

Section S1. Intervention Descriptions, Patient Flow Diagrams and Descriptive Statistics of Providers
The interventions analyzed in this report are described below.

1. *Vision Centers* are permanent, primary eye health facilities that provide diagnoses for refractive error, cataracts and other eye health problems. Typically serving a catchment of 50,000 people, these facilities are designed to be the first point of interface for eye health services in the community. Almost all vision centers in the providers' networks had the capacity to dispense spectacles immediately. Additionally, each facility is linked to a secondary or tertiary hospital for referral. While the main services are common across providers (i.e. refraction and surgery referral), organizations adopt slightly different operating models for their centers, such as the extent of internal training, the number of staff at each center, and the types of clinical equipment used. These differences have been described in detail elsewhere.[1]
2. *Eye camps* have been a prominent intervention for identifying individuals with cataracts and visual impairment over several decades in India. Though eye camps can be either one-off or have fixed, recurrent schedules, the distinguishing feature is its non-permanent nature. Promotion occurs in the lead up to the camp to increase awareness, and on the given day, doctors and technicians visit a community with clinical equipment to screen and diagnose URE, cataracts and other eye conditions in a dedicated space. Most providers provide free spectacles either on the day or arrange delivery to the patient within a few weeks. Cataract surgery, including travel, is typically subsidized.
3. *Door-to-door screening* is a relatively new mode of case finding for providers, and was adopted by some as a response to reduced movement of the general population during the COVID-pandemic. This strategy entails community health workers visiting households and screening for eye conditions at homes. Individuals who are determined to have poor visual acuity are referred to a vision center for further diagnostics. Those who are determined to have cataracts are referred to a hospital for surgery, often with travel subsidized.
4. *School eye screening* involves screening children for URE and other eye conditions during school hours. Visits by doctors and technicians are planned with the school management, and a dedicated day is devoted to the screening activities. Only one provider trained teachers to conduct preliminary screening, with the remainder using teachers for solely administrative purposes. Children with URE are provided glasses within a week, and those with more serious eye conditions are referred to a hospital for further diagnostics.
5. *Teleophthalmology* refers to the use of technology that allows doctors at a location other than the vision center to diagnose patient eye conditions. This is achieved via live consultations and the transmission of clinical data, including high resolution eye scans. In our study, only two providers used teleophthalmology in vision centers during the period of analysis, while a third had begun implementation in July 2021.

Table S1.1: Description of provider activities and cost drivers for major categories of cost by intervention type

	**Planning and preparation**	**Equipment**	**Human Resources**	**Other expenses**
**Vision Centers**	Activities include identifying an appropriate site for a facility, renting a building, fitting out the building with the necessary furniture and optical displays, and promoting the opening of the vision center through community engagement.	Each vision center is supplied with the necessary clinical equipment to provide eye health services. All vision centers have the capability to provide refractive services and screening for cataracts. Only some providers had the capability to examine the retina and optic nerve. Equipment includes slit lamps, streak retinoscopes, auto refractometers, vision drums and in some cases fundus cameras. General clinical equipment includes thermometers, blood pressure apparatus and consumables.<br><br>Each vision center also includes the necessary IT equipment such as computers, modems and patient management software.	Each vision center typically has two employees – a vision technician to perform clinical work and an administrator to provide admin support, patient management, health promotion and sales.<br><br>Training was provided by all NGOs, though the exact mode differed by provider. Some NGOs delivered multi-year training courses with the intent of placing graduates into vision centers. Others hired already trained personnel from the labor market and provided only days of training to familiarize new employees with the providers' systems and processes.	Other expenses include rent, utilities, equipment maintenance and staff travel.
**Eye Camps**	In preparation for eye camps, staff from the provider first engage local actors from community groups, NGOs or village health depots. Substantial effort is made to promote the eye camps using flyers, signs and door-to-door awareness raising.	Equipment used at eye camps includes portable slit lamps, vision drums, handheld autorefractometers, streak retinoscopes and ophthalmoscopes.	While the number of staff required per camp varies by provider, it typically includes one or more optometrists and health technicians, a counsellor, administrative support staff, and drivers.	The largest expense in this category is typically subsidized transport costs for those diagnosed with cataracts. Other costs include refreshments and, travel / accommodation costs for staff who may need to stay overnight in remote areas.
**School Eye Screening**	The main activity is to engage school administrators, and plan for school visits by the screening team. Some providers train teachers to do preliminary screening before the school visit.	Equipment used during school screening includes vision drum, handheld autorefractometer, streak retinoscope and ophthalmoscope. IT equipment includes tablet, laptops and portable wifi.	While the number of staff required per camp varies by provider, it typically includes one paediatric optometrist, one or more health technicians, a counsellor, administrative support staff, and drivers.	Costs include staff travel costs and per diems.
**Door-to-Door**	The primary activity is to identify a community for screening. Most providers used already employed health technicians, though one provider hired a dedicated health worker from the community to undertake screening.	Equipment varied by provider. Some providers used basic equipment such as visual acuity card, a measuring tape and a torchlight. One provider used an electronic, tablet-based diagnosis tool. Another provider used streak retinoscope and an ophthalmoscope.	Door-to-door screening was typically conducted by a team of 4-6 health workers that were each allocated a certain number of households within a catchment area. Support staff accompanied the screening team.	The largest expense in this category is typically subsidized transport costs for those diagnosed with cataracts.
**Teleophthalmology**	Activities include developing appropriate communication and IT systems, training vision center staff and	Equipment typically includes cameras, upgraded patient software and internet connections with greater bandwidth.	The main cost in this category are the costs of doctors to act as teleconsultants for vision centers.	Other expenses include telecommunications subscriptions with greater bandwidth and higher internet speeds, equipment maintenance.

	developing appropriate clinical protocols.			

Figure S1.1: Patient flow diagrams

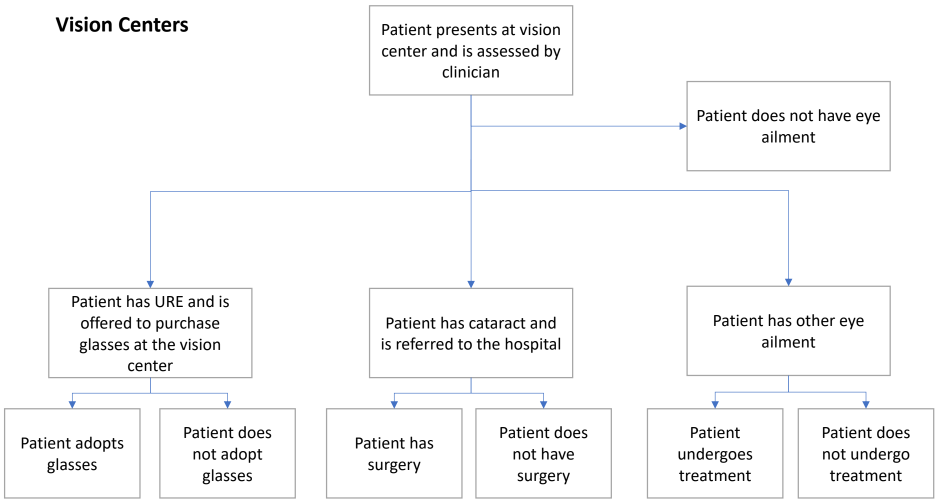

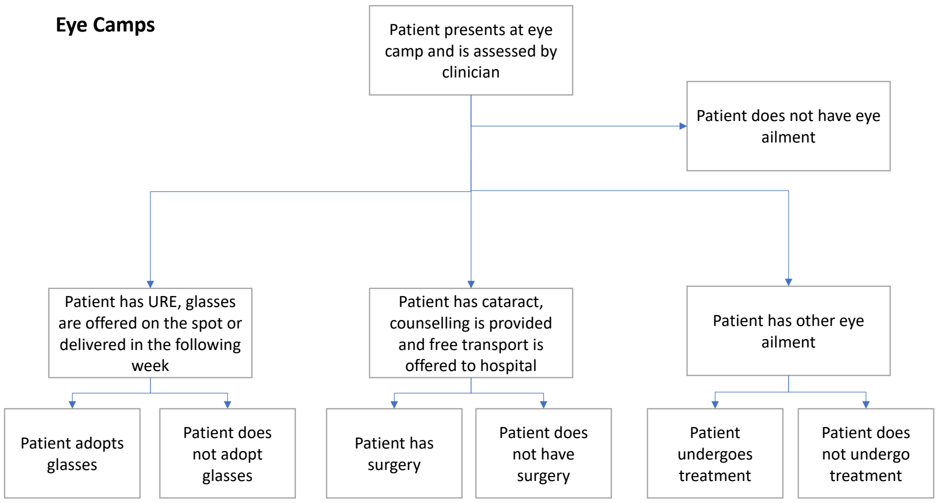

School Eye Screening

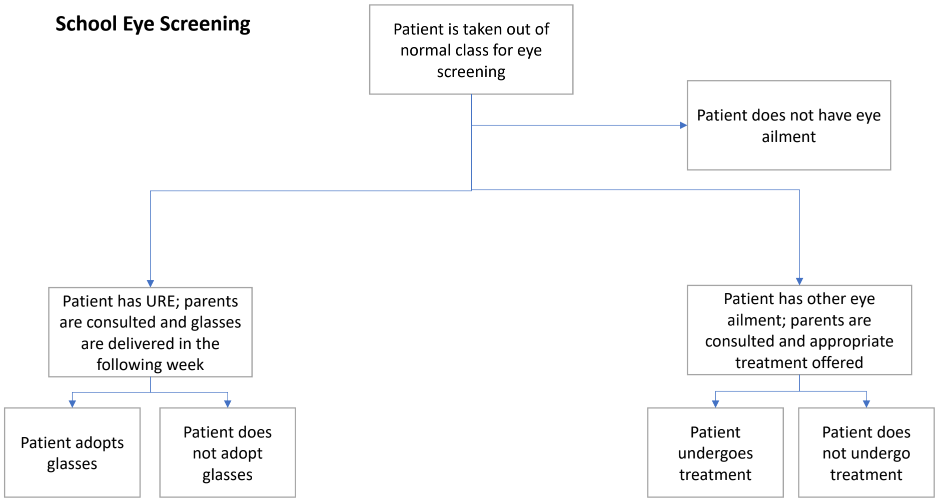

Door-to-Door

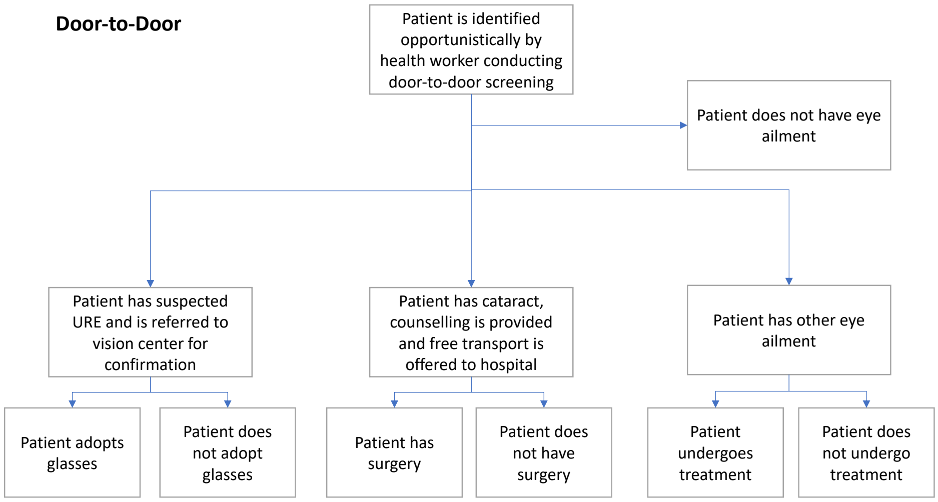

Table S1.2: Descriptive Statistics, Provider Data Financial Year 2019-20

**Provider**	Geographic scope of operations	Number of vision centers in network	Number of people screened in vision centers	Number of people screened in eye camps	Number of children screened via school screening	Number of people screened via door-to-door
Aravind	Tamil Nadu, Andhra Pradesh, Pondicherry, Karnataka, Kerala	79	688,566	94,889	61,647	-
HVD	Maharashtra	4	30,424	21,373	33,376	-
LVPEI	Andhra Pradesh, Telangana, Odisha, Karnataka	184	386,992	67,177	42,261	73,878
SNC	Uttar Pradesh	42	131,050	32,479	111,829	17,390
SCEH	Delhi	34	87,600	38,244	-	175,057
VMANNN	West Bengal	12	49,293	53,556	83,124	20,327
**TOTAL**		**355**	**1,373,925**	**307,718**	**332,237**	**286,652**

Notes: All data were provided for the financial year 2019-20, except for door-to-door screening where data is from most recent year available. School screening data for SNC and VMANNN were based on multi-year projects that ran until 2019, and represent average children screened per year. The total number of people screened in eye camps from Aravind represents only camps around the Madurai hospital (for which cost data was available). The total number of people screened in all Aravind camps was 354,183. Providers are Aravind Eye Care System (Aravind), H.V. Desai Eye Hospital (HVD), LV Prasad Eye Institute (LVPEI), Sadguru Netra Chikitsalaya (SNC), Dr. Shroff's Charity Eye Hospital (SCEH) and Vivekananda Mission Asram Netra Niramay Niketan (VMANNN).

Section S2. Methods and Data Collection Detail

The aim of the exercise was to collect the full financial costs of the strategies. Full (as opposed to incremental) costs were chosen since we wished to assess the most complete picture of costs in the initial stage, with incremental cost-effectiveness analysis to be conducted after data collection. Moreover, given the relatively limited provision of primary eye health services in India (e.g. a likely 90% shortfall in the number of vision centers), full costs are more useful for budgeting future scale up of strategies. Financial (as opposed to economic) costs were chosen since providers had a more precise view of financial costs.

Data was collected via a spreadsheet template with data collection occurring between June and September 2021. The template requested general administrative data including the number of patients screened, the number of patients diagnosed with URE, cataracts or other eye conditions, and the number of patients who acted on those diagnoses with spectacles, surgery or other remedial actions. We also requested the number and unit costs of inputs used for each of the interventions over the specified time period. This included the estimated number of days of work for preparation, planning and operations, salaries of relevant staff, the cost and number of various pieces of clinical equipment, IT equipment and consumables used and, the costs of other operating expenses such as travel, training, electricity and rent. The procurement costs of spectacles were also obtained for the cost-effectiveness analysis. All equipment costs were annualized over their expected life at a 3% discount rate. We assumed that furniture and specialized clinical equipment such as slit lamp and fundus camera had a useful life of 8 years based on discussions with the providers. Electronic equipment such as laptops, tablets were assumed to last 3 years, while general clinical equipment such as thermometers and glucometers were assumed to last 2 years before replacement.

Semi-structured interviews were conducted between the lead authors (BW and KS) and the providers' financial and operational teams responsible for providing data. At least one conversation was had before the template was completed to describe the process and required outcomes. After the template had been filled in and delivered to the lead authors, the data were checked for errors, and consistency with other providers. Thereafter, at least one, but often several further conversations were had with each provider to clarify any questions. One important line of questioning was to ensure consistency between providers in the type of costs considered. If a provider did not include a cost that other providers had included, a reason was sought and noted, or additional data requested. Cost categories and sub-categories are presented in Tables S3.1-S3.5.

Section S3. Probabilistic Analysis: Cost Analysis

Probabilistic analysis was conducted by varying each cost category over 10,000 Monte Carlo simulations, and then summing to identify total costs. Each cost was assumed to follow a gamma distribution following a recent economic modelling study of eye health screening in China.[2] Parameters for the gamma distribution were set to minimize the distance between the minimum provider estimate and the 10th percentile value, and the maximum provider estimate and the 90th percentile value. Details of the gamma distributions are presented in Tables S3.1-S3.5. There was insufficient information to estimate gamma distributions for teleophthalmology by individual costs, so we only varied total costs following a gamma distribution with parameters alpha = 2.3 and beta = 555.9.

Table S3.1: Cost hierarchy for Vision Centers and parameters for Monte Carlo Simulation

Cost Category	Subcategory	Distribution parameters for Monte Carlo Simulation
Planning and preparation	Planning	Gamma(5.2,4.44)
	Site Preparation incl. Furniture + Fixtures	Gamma(2.94,143.02)
	Community Engagement and Promotion	Gamma(0.81,105.05)
	Specialist Training	Gamma(8.7,50.43)
Equipment	Clinical equipment for assessing the cornea and assessing visual acuity	Gamma(3.98,268.84)
	Clinical equipment for assessing the retina and optic nerve	Gamma(5.19,98.09)
	General medical equipment	Gamma(2.69,55.58)
	IT equipment	Gamma(2.67,176.96)
Human Resources	Eye Technician	Gamma(5.67,525.98)
	Other Support Staff (Administrator, Outreach Worker)	Gamma(42.94,59.52)
Other Operating Expenses	Travel	Gamma(1.11,613.84)
	Rent	Gamma(9.48,146.57)
	Utilities	Gamma(10.84,35.81)
	Miscellaneous	Gamma(2.49,211.96)

Table S3.2: Cost hierarchy for Teleophthalmology

Cost Category	Subcategory
Planning and preparation	Planning, preparation and training
Equipment	Additional IT equipment and software
Human Resources	Additional doctor time
Other Operating Expenses	Additional internet costs

Table S3.3: Cost hierarchy for School Screening and parameters for Monte Carlo Simulation

Cost Category	Subcategory	Distribution parameters for Monte Carlo Simulation
Planning and preparation	Planning	Gamma(1.81,1026.8)
	School Engagement	Gamma(1.79,1079.37)
Equipment	Clinical equipment	Gamma(1.6,5358.52)
	IT and other equipment	Gamma(1.15,3457.9)
Human Resources	Clinical Staff	Gamma(6.96,5481.4)
	Support Staff	Gamma(1,16690.72)
Other Operating Expenses	Travel	Gamma(1.51,7810.04)
	Miscellaneous	Gamma(3.1,984.1)

Table S3.4: Cost hierarchy for Eye Camps and parameters for Monte Carlo Simulation

Cost Category	Subcategory	Distribution parameters for Monte Carlo Simulation
Planning and preparation	Planning	Gamma(0.92,41656.33)
	Community Engagement	Gamma(1.37,58437.43)
Equipment	Clinical equipment	Gamma(0.47,38961.17)
Human Resources	Clinical Staff	Gamma(2.29,29171.01)
	Support Staff	Gamma(1.37,24560.59)
Other Operating Expenses	Staff Travel	Gamma(1.49,31062.69)
	Miscellaneous	Gamma(0.5,34088.01)
	Subsidized Patient Travel Costs	Gamma(1.02,81621.65)

Table S3.5: Cost hierarchy for Door-to-door screening and parameters for Monte Carlo Simulation

Cost Category	Subcategory	Distribution parameters for Monte Carlo Simulation
Planning and preparation	Planning	Gamma(1.77,809.91)
	Community Engagement	Gamma(0.68,18777.9)
Equipment	Clinical equipment	Gamma(4.18,1009.03)
Human Resources	Clinical Staff	Gamma(4.88,6034.2)
	Support Staff	Gamma(0.15,21611.64)
Other Operating Expenses	Staff Travel	Gamma(1.39,8534.5)
	Miscellaneous	Fixed at average value
	Subsidized Patient Travel Costs	Gamma(0.19,158400.05)

Section S4. Detailed Costing Formulas and Results

Formulas

For all interventions, the formulas for calculating URE and cataract case finding and treatment initiation costs are:

$$CFTI_{URE} = \frac{Costs\ attributable\ to\ URE\ case\ finding\ and\ treatment\ inititation}{Number\ of\ spectacles\ provided}$$

$$CFTI_{Cataract} = \frac{Costs\ attributable\ to\ cataract\ case\ finding\ and\ treatment\ inititation}{Number\ of\ cataract\ surgeries\ performed}$$

The numerators in each equation vary by intervention and are presented below.

For vision centers:

$$Costs\ attributable\ to\ URE\ case\ finding\ and\ treatment\ initiation = \begin{pmatrix} Planning\ and\ prep\ cost + clinical\ equipment\ cost + human\ resource\ cost \\ + other\ operating\ expenses \\ - clinical\ equipment\ for\ asessing\ the\ retina\ and\ optic\ nerve\ cost \end{pmatrix} * \frac{URE\ diganosed}{Total\ diagnoses}$$

$$Costs\ attributable\ to\ cataract\ case\ finding\ and\ treatment\ initiation = \begin{pmatrix} Planning\ and\ prep\ cost + clinical\ equipment\ cost + human\ resource\ cost \\ + other\ operating\ expenses \\ - clinical\ equipment\ for\ asessing\ the\ retina\ and\ optic\ nerve\ cost \end{pmatrix} * \frac{Cataracts\ diganosed}{Total\ diagnoses}$$

In the case of vision centers all costs are apportioned to URE or cataracts based on their share in total diagnoses. The exception is clinical equipment for assessing the retina and optic nerve. Since this equipment is not required to diagnose URE or cataracts, these costs are assigned to other ailments.

For eye camps:

$$Costs\ attributable\ to\ URE\ case\ finding\ and\ treatment\ initiation = \begin{pmatrix} Planning\ and\ prep\ cost + clinical\ equipment\ cost + human\ resource\ cost \\ + other\ operating\ expenses \\ - subsidized\ patient\ travel\ costs \end{pmatrix} * \frac{URE\ diganosed}{Total\ diagnoses}$$

$$Costs\ attributable\ to\ cataract\ case\ finding\ and\ treatment\ initiation = \begin{pmatrix} Planning\ and\ prep\ cost + clinical\ equipment\ cost + human\ resource\ cost \\ + other\ operating\ expenses \\ - subsidized\ patient\ travel\ costs \end{pmatrix} * \frac{Cataracts\ diganosed}{Total\ diagnoses} + subdized\ patient\ travel\ costs$$

In the case of eye camps, all costs are apportioned to URE or cataracts based on their share in total diagnoses. The exception is subsidized patient travel costs which are provided for cataract surgery. Therefore, these are completely apportioned to costs attributable to cataract case finding and treatment initiation.

For door-to-door screening:

$$Costs\ attributable\ to\ URE\ case\ finding\ and\ treatment\ initiation = \begin{pmatrix} Planning\ and\ prep\ cost + clinical\ equipment\ cost + human\ resource\ cost \\ + other\ operating\ expenses \\ - subsidized\ patient\ travel\ costs \end{pmatrix} * \frac{URE\ diganosed}{Total\ diagnoses} + vision\ center\ follow\ up\ costs$$

$$\begin{aligned}&Costs\ attributable\ to\ cataract\ case\ finding\ and\ treatment\ initiation\\&= \begin{pmatrix}Planning\ and\ prep\ cost + clinical\ equipment\ cost + human\ resource\ cost\\ + other\ operating\ expenses\\ - subsidized\ patient\ travel\ costs\end{pmatrix} * \frac{Cataracts\ diganosed}{Total\ diagnoses}\\&+ subdized\ patient\ travel\ costs\end{aligned}$$

In the case of door-to-door screening, as with camps, subsidized patient travel costs is wholly assigned to costs attributable to cataract case finding and treatment initiation. For costs attributable to URE case finding and treatment initiation, follow up costs at the vision center are added.

For school-screening:

$$\begin{aligned}&Costs\ attributable\ to\ URE\ case\ finding\ and\ treatment\ initiation\\&= \begin{pmatrix}Planning\ and\ prep\ cost + clinical\ equipment\ cost + human\ resource\ cost\\ + other\ operating\ expenses\end{pmatrix} * \frac{URE\ diganosed}{Total\ diagnoses}\end{aligned}$$

School screening only case finding and treatment initiation costs for URE are considered.

Detailed Results

Costs and sub-category costs of each intervention and 95% confidence intervals are presented below.

Table S4.1: Vision Centers, Costs per 100,000 screened (2020 USD)

Category	Sub Category	Point estimate	Lower bound 95% CI	Upper bound 95% CI
Planning and preparation	Planning (A)	598	203	1,212
	Site Preparation incl. Furniture + Fixtures (B)	10,890	2,252	26,285
	Community Engagement and Promotion (C)	2,223	28	9,044
	Specialist Training (D)	11,338	5,107	20,054
Equipment	Clinical equipment for assessing the cornea and visual acuity (E)	27,656	7,421	60,821
	Clinical equipment for assessing the retina and optic nerve (F)	13,174	4,441	26,526
	General medical equipment (G)	3,877	719	9,632
	IT equipment (H)	12,239	2,221	30,161
Human Resources	Eye Technician (I)	77,137	27,078	152,031
	Other Support Staff (Administrator, Outreach Worker) (J)	66,053	47,849	87,420
Other Operating Expenses	Travel (K)	17,661	641	62,959
	Rent (L)	35,919	17,091	61,949
	Utilities (M)	10,039	4,990	16941
	Miscellaneous (N)	13,680	2,284	35036
**TOTAL**	**Total case finding and treatment initiation costs (O=A+B+C+D+E+F+G+H+I+J+K+L+M+N)**	302,485	225,355	400,289
Diagnoses shares	URE diagnosed (as % of all diagnoses) (P)	66%		
	Cataracts diagnosed (as % of all diagnoses) (Q)	22%		
	Other eye ailments diagnosed (as % of all diagnoses) (R)	12%		
Outcomes	Spectacles provided per 100,000 screened (S)	17,574		
	Cataract surgeries performed per 100,000 screened (T)	5,409		
Results	Case finding and treatment initiation costs, URE (U=(O-F)*P/S)	10.8	8.0	14.4
	Case finding and treatment initiation costs, Cataracts (V=(O-F)*Q/T)	11.9	8.8	15.9

Table S4.2: Eye camps, Costs per 100,000 screened (2020 USD)

Cost Category	Sub Category	Point estimate	Lower bound 95% CI	Upper bound 95% CI
Planning and preparation	Planning (A)	38,545	831	148,982
	Community Engagement (B)	80,609	4,911	258,553
Equipment	Clinical equipment (C)	18,585	12	95,256
Human Resources	Clinical Staff (D)	66,876	9,872	174,521
	Support Staff (E)	33,718	1,910	109,039
Other Operating Expenses	Staff Travel (F)	46,370	3,237	142,039
	Miscellaneous (G)	17,198	20	85,241
	Subsidized Patient Travel Costs (H)	83,840	2,165	297,676
**TOTAL**	**Total case finding and treatment initiation costs (I = A+B+C+D+E+F+G+H)**	385,742	170,111	699,504
Diagnoses shares	URE diagnosed (as % of all diagnoses) (J)	46%		
	Cataracts diagnosed (as % of all diagnoses) (K)	53%		
	Other eye ailments diagnosed (as % of all diagnoses) (L)	2%		
Outcomes	Spectacles provided per 100,000 screened (M)	17,329		
	Cataract surgeries performed per 100,000 screened (N)	17,752		
Results	Case finding and treatment initiation costs, URE (O=(I-H)*J/M)	8.0	3.4	14.4
	Case finding and treatment initiation costs, Cataracts (P=((I-H)*K+H)/N)	13.7	5.6	27.0

Table S4.3: Door-to-Door, Costs per 100,000 screened (2020 USD)

Cost Category	Sub Category	Point Estimate	Lower bound 95% CI	Upper bound 95% CI
Planning and preparation	Planning (A)	1,440	149	4,197
	Community Engagement (B)	12,772	74	55,169
Equipment	Clinical equipment (C)	4,227	1,188	9,232
Human Resources	Clinical Staff (D)	29,491	9,362	61,348
	Support Staff (E)	3,415	0	28,595
Other Operating Expenses	Staff Travel (F)	11,945	781	37,965
	Miscellaneous (G)	1,991	1,991	1,991
	Subsidized Patient Travel Costs (H)	31,650	0	234,817
	Vision Center Follow Up for URE (I)	202,816	na	na
**TOTAL**	**Total case finding and treatment initiation costs (J = A+B+C+D+E+F+G+H+I)**	299,748	243,637	511,093
Diagnoses shares	URE diagnosed (as % of all diagnoses) (K)	67%		
	Cataracts diagnosed (as % of all diagnoses) (L)	30%		
	Other eye ailments diagnosed (as % of all diagnoses) (M)	3%		
Outcomes	Spectacles provided per 100,000 screened (N)	9,563		
	Cataract surgeries performed per 100,000 screened (O)	4,543		
Results	Case finding and treatment initiation costs, URE (P=((J-H-I)*K+I)/M)	25.8	24.1	30.7
	Case finding and treatment initiation costs, Cataracts (Q=((J-H-I)*L+H)/O)	11.3	2.2	56.2

Table S4.4: School Eye Screening, Costs per 100,000 screened (2020 USD)

Cost Category	Sub Category	Point Estimate	Lower bound 95% CI	Upper bound 95% CI
Planning and preparation	Planning (A)	1,865	181	5,283
	School Engagement (B)	1,934	199	5,684
Equipment	Clinical equipment (C)	8,597	723	26,492
	IT and other equipment (D)	4,003	154	13,753
Human Resources	Clinical Staff (E)	38,202	15,400	71,694
	Support Staff (F)	16,808	448	63,083
Other Operating Expenses	Travel (G)	11,795	871	36,819
	Miscellaneous (I)	3,059	657	7,244
**TOTAL**	**Total case finding and treatment initiation costs (J = A+B+C+D+E+F+G+H+I)**	86,262	45,555	146,067
Diagnoses shares	URE diagnosed (as % of all diagnoses) (K)	84%		
	Cataracts diagnosed (as % of all diagnoses) (L)	0%		
	Other eye ailments diagnosed (as % of all diagnoses) (M)	16%		
Outcomes	Spectacles provided per 100,000 screened (N)	2,459		
	Cataract surgeries performed per 100,000 screened (O)	-		
Result	Case finding and treatment initiation costs, URE (P=(J*K/N)	29.3	15.5	49.6

Facility level costs for Vision Centers

Table S4.5: Vision Center Costs, per facility

Cost Category	Sub Category	Cost per vision center ($)	Lower bound 95% CI	Upper bound 95% CI
Planning and preparation	Planning	23.13	7.84	46.90
	Site Preparation incl. Furniture + Fixtures	421.46	87.16	1,017.30
	Community Engagement and Promotion	86.03	1.07	350.01
	Specialist Training	438.82	197.66	776.14
Equipment	Clinical equipment for assessing the cornea and visual acuity	1,070.36	287.20	2,353.92
	Clinical equipment for assessing the retina and optic nerve	509.86	171.86	1,026.60
	General medical equipment	150.05	27.83	372.79
	IT equipment	473.69	85.97	1,167.28
Human Resources	Eye Technician	2,985.37	1,047.99	5,883.90
	Other Support Staff (Administrator, Outreach Worker)	2,556.38	1,851.84	3,383.34
Other Operating Expenses	Travel	683.50	24.80	2,436.63
	Rent	1,390.13	661.47	2,397.55
	Utilities	388.54	193.12	655.66
	Miscellaneous	529.46	88.39	1,355.96
**TOTAL**	**TOTAL**	11,706.80	8,721.73	15,492.02
Spectacles	Spectacles Cost	4.77	3.35	6.48
**TOTAL with Spectacles**	**TOTAL with Spectacles**	14,953.51	11,781.92	18,912.56

Section S5. Teleophthalmology Costs and Scenario Analysis

Two providers were able to identify incremental costs that were attributable to teleophthalmology during FY 2019-2020. Another provider had recently implemented teleophthalmology in July 2021. Using input from these three providers we estimated the costs of installing teleophthalmology for one vision center.

While providers noted several key advantages from teleophthalmology including reduced visits to hospital outpatient departments, improved diagnoses and increased uptake of medical advice, they were unable to precisely attribute how much teleophthalmology improved outcomes along these factors. To assess the potential cost-effectiveness of teleophthalmology we therefore conduct a scenario analysis to determine how much of the 'initiation gap' - i.e. the difference between spectacles and cataract surgeries advised, and actually initiated – would need to be closed by teleophthalmology for it to incur similar effective case finding and treatment initiation costs as the remaining strategies for the providers which did not adopt teleophthalmology. This analysis assumes that one plausible benefit of teleophthalmology is greater patient trust in diagnoses, leading to higher uptake of glasses or surgery. Across all these vision centers the initiation gap for spectacles was 340 per vision center, while for cataracts it was 152. To illustrate, a 10% closure of the initiation gap would therefore equal 34 more spectacles dispensed and 15·2 more cataracts performed.

The results of the scenario analysis are depicted in Figure S5.1 for both spectacles and cataract surgeries across initiation closure rates from 10-100%. For comparison, dotted lines indicate the range between USD 8·0 and 14·0 – the range of case finding and treatment initiation costs from alternative strategies focusing on the general population (vision centers and eye camps, and door to door screening for cataracts). The results show that teleophthalmology would need to close 16-27% of the initiation gap for spectacles and 17-29% of the initiation gap for cataract surgeries for it to be equally as cost-effective as alternative strategies.

Figure S5.1: Scenario analysis of teleophthalmology's impact on case finding and treatment initiation costs

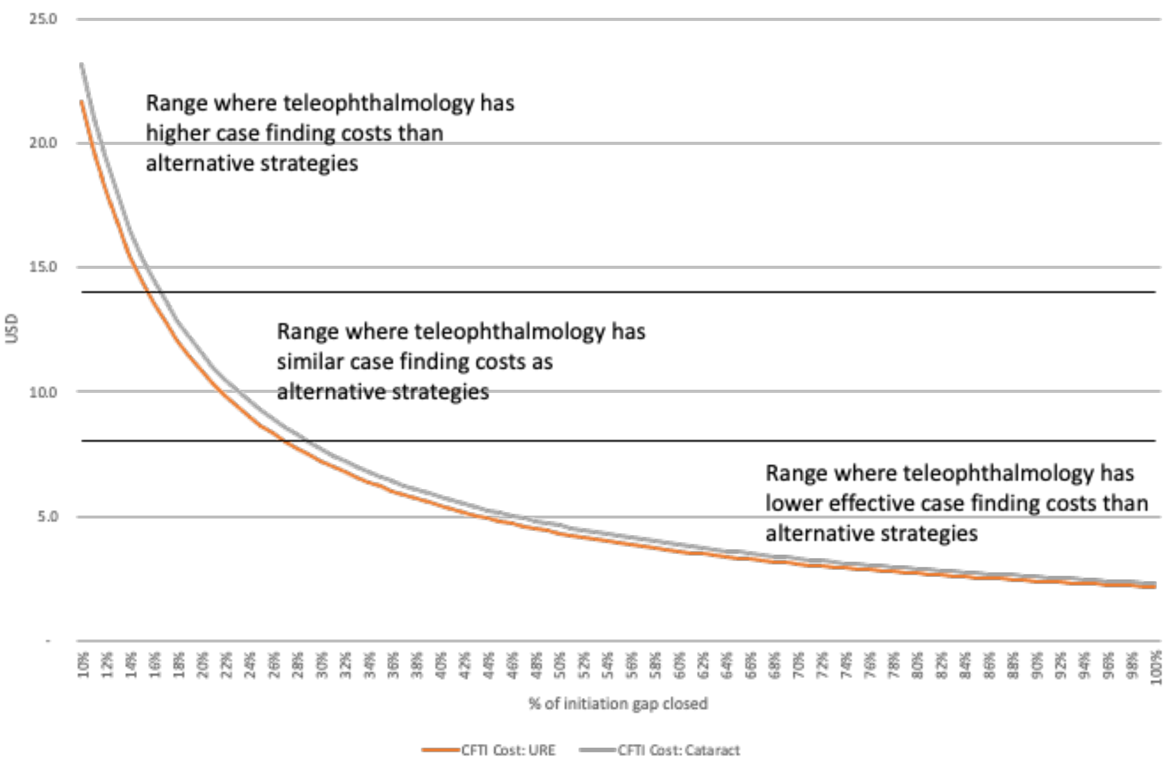

Section S6. Cost-effectiveness analysis

Primary Scenario
Incremental cost-effectiveness ratios for intervention $i \in I$ (eye camps, vision centers, door-to-door, school screening) are estimated according to the following formula:

$$ICER_i = {(Cost_i - Cost_{comparator})}\Big/{(DALY_i - DALY_{comparator})}$$

Where the subscript *comparator*, represents the costs and DALYs averted in the comparison scenario, either the baseline standard of care or the next least costly intervention. Costs are estimated from a societal perspective.

Costs can be separated for URE and cataracts:

$$Cost_i = Cost_{i,URE} + Cost_{i,cat}$$

Where:

$$Cost_{i,URE} = Cost_{i,CFTI\ URE} + Cost_{i,spectacles} + Cost_{i,patient\ costs\ URE}$$

$$Cost_{i,cat} = Cost_{i,CFTI\ cat} + Cost_{i,surgery} + Cost_{i,patient\ costs\ cat}$$

Here subscripts denote costs of case finding and treatment initiation (CFTI), treatment (spectacles or surgery) and patient costs for accessing services.

Case finding and treatment initiation costs are estimated from provider data. The costs of spectacles ($4.8) are also estimated from provider procurement data. The costs of cataract surgery are assumed to be $71, the reimbursement cost provided by the government of India under the national insurance scheme. Patient costs for visiting vision centers include travel, waiting time and incidentals are sourced from a study by Kovai and colleagues.[3] Patient costs associated with cataract surgery include travel and lost productivity and are sourced from a study by Le and colleagues.[4]

DALYs averted from intervention *i* are estimated according to the following formula:

$$DALY_i = DALY_{i,URE} + DALY_{i,cat}$$

Where:

$$DALY_{i,URE} = K_{i,spectacles} * DALY\ per\ case\ URE$$
$$DALY_{i,cat} = K_{i,surgery} * DALY\ per\ case\ cat$$

Here *K* denotes the number of spectacles provided or cataract surgeries performed from intervention *i*. And lastly:

$$DALY\ per\ case\ URE = \sum_{t=1}^{j} \frac{\sum_{n \in N}(w_n * p_{n,URE})}{(1+r)^{t,URE}}$$

$$DALY\ per\ case\ cat = \sum_{t=1}^{j} \frac{\sum_{n \in N}(w_n * p_{n,cat})}{(1+r)^{t,cat}}$$

Here *w* denotes the disability weight for four visual acuity states $n \in N$ (mild, moderate, severe and blind), and *p* is the population prevalence for each state-disease combination. The discount rate is *r* and *j* is the longevity of benefits from the intervention.

Disability weights are from the Global Burden of Disease i.e. blindness (0·187), severe visual impairment (0·184), moderate visual impairment (0·031) and mild visual impairment (0·003).[5] The distribution of visual

acuity is from the National Blindness and Visual Impairment Survey.[6] For URE the population prevalence across visual acuity states is blind = 0.01%; severe = 0.19%; moderate = 15.49%, mild = 84.30%. For cataract it is blind = 9.72%, severe = 11.85%, moderate = 53.95%, mild = 24.48%. Note that our DALY calculations do not include DALYs from years of life lost[7] implying that cost per DALY results represent a conservative upper bound. Moreover, the DALYs averted per year from cataracts is likely an underestimate since by adopting the current state of visual acuity distribution (where most people with cataracts only suffer from moderate or early visual impairment), we implicitly assume that cataracts would not worsen over time.

The longevity of benefits for addressing URE is 3 years, the estimated useful life of a pair of glasses. The longevity of benefit for addressing cataract is 19.9 years the average life expectancy from those suffering from cataracts as estimated by Wong and colleagues.[8]

Alternative Baseline Specifications
In the primary scenario we assume that the baseline, standard of care is one of no intervention i.e. in the absence of the intervention, individuals would not have their URE or cataracts treated. Reasons for this approach are provided in the main text. To the best of our knowledge there is no credible evidence in the Indian context that would allow for a more precise estimation of the baseline, standard of care. To test the importance of this assumption, we assess different baseline case finding rates b, where b = 10%, 20% and 30% of URE and cataracts would otherwise be treated. We assume that these individuals would self-present at provider hospitals for treatment. In these instances, $DALY_{base}$ is straightforwardly estimated as $\left[\sum_{i \in I} DALY_i\right] * b$. For $Cost_{base}$, we note that those who would otherwise be treated would still incur costs of surgery or glasses and patient costs. The only costs that would not be incurred are case finding and treatment initiation costs since the individuals self-present at hospitals. Here the relevant formula is:

$$Cost_{base} = \sum_{i \in I} (Cost_i - Cost_{i,CFTI\ URE} - Cost_{i,CFTI\ cat}) * b$$

Sensitivity Analyses
We perform one-way and probabilistic sensitivity analyses varying parameters. For the one-way sensitivity analyses we vary case finding and treatment initiation costs using lower and upper ends of the 95% confidence intervals. Discount rates are assumed to be 0% at the low end, and 8% at the high end. Costs of surgery take values of \$42.6 and \$106.5 based on the reimbursement cost provided by Seva Foundation to providers and the approximate costs of surgery noted in a study from Southern India.[4] Spectacles cost range from \$3.3 and \$6.5 based on 95% confidence intervals for that cost category. Patient costs of surgery and vision centers take the low and upper values of confidence intervals from the relevant studies.[3,4] Alternative baseline specifications are also assessed using the methodology presented above.

For the probabilistic sensitivity analysis, the same parameters are varied across 10,000 Monte Carlo simulations. Case finding and treatment initiation as well as spectacles costs are based on Monte Carlo simulations noted previously. DALY discount rates vary uniformly from 0% to 8%. Costs of surgery vary uniformly from \$42.6 to \$106.5. Patient costs vary as a Gaussian distribution based on reported confidence intervals from the relevant studies.[3,4] Alternative baseline case finding rates are assumed to vary uniformly between 0 and 30%.

Results are presented in Table S6.1. Overall, addressing cataracts has a lower cost per DALY than addressing URE, so interventions that have a higher share of outcomes related to cataracts are more cost effective.

Table S6.1: Cost Effectiveness Analysis of Four Interventions – Main Results

	Vision Center	Camps	Door-to-Door	School Eye Screening	Baseline Scenario 10%	Baseline Scenario 20%	Baseline Scenario 30%
URE							
Spectacles provided	241,454	53,325	27,413	8,169	33,036	66,072	99,108
Case finding cost (USD)	2,614,171	424,860	706,392	239,309	-	-	-
Cost of spectacles (USD)	1,152,580	254,545	130,855	38,994	157,697	315,395	473,092
Patient costs (USD)	295,721	-	33,574	-	32,930	65,859	98,789
**Total costs for providing spectacles to address URE (USD)**	**4,062,473**	**679,405**	**870,821**	**278,303**	**190,627**	**381,254**	**571,881**
Longevity of benefit (years)	3.0	3.0	3.0	3.0	3.0	3.0	3.0
DALYS averted from addressing URE	5,266	1,163	598	178	720	1,441	2,161
Total costs / Total DALYs – URE (USD)	772	584	1,457	1,562	265	265	265
Cataracts							
Cataract surgeries done	74,311	54,627	13,024	-	14,196	28,392	42,589
Case finding cost (USD)	884,617	746,404	146,531	-	-	-	-
Cost of surgery (USD)	5,276,271	3,878,657	924,714	-	1,007,964	2,015,928	3,023,893
Patient costs (USD)	2,070,508	1,522,058	362,875	-	395,544	791,088	1,186,632
**Total costs for cataract surgery (USD)**	**8,231,396**	**6,147,119**	**1,434,120**	**-**	**1,403,508**	**2,807,017**	**4,210,525**
Longevity of benefit (years)	19.9	19.9	19.9	-	19.9	19.9	19.9
DALYS averted from addressing cataracts	63,169	46,436	11,071	-	12,068	24,135	36,203
Total costs / Total DALYs – Cataracts (USD)	130	132	130	na	116	116	116
URE + Cataracts							
Total costs (USD)	12,293,868	6,826,524	2,304,941	278,303	1,594,135	3,188,271	4,782,406
Total DALYs avoided	68,435	47,599	11,669	178	12,788	25,576	38,364
Total costs / Total DALYs (USD)	**180**	**143**	**198**	**1,562**	**125**	**125**	**125**

Notes: Values are presented in 2020 USD. Columns report results for each intervention (columns 2-5) plus three alternative baseline scenarios where 10%, 20% and 30% of individuals with URE and cataract are assumed to self-present at hospitals (columns 6-8). Total costs / total DALYs are not equivalent to incremental cost-effectiveness ratios since they do not include a comparator intervention.

Table S6.2 presents the results of the one-way sensitivity analyses. For all specifications, camps remain the most cost-effective, followed by vision centers, door-to-door and finally school screening, suggesting our findings are robust to different assumptions of costs, discount rates and counterfactual conditions.

Table S6.2: One-way Sensitivity Analyses, Cost per DALY

	**Camps**	**Vision Centers**	**Door-to-Door**	**School Screening**
**Primary Scenario**	143	262	Weakly dominated	Weakly dominated
CFTI low end of 95% CI	129	251	Weakly dominated	Weakly dominated
CFTI upper end of 95% CI	166	267	Weakly dominated	Weakly dominated
DALYs discounted at 0%	107	204	Weakly dominated	Weakly dominated
DALYs discounted at 8%	215	369	Weakly dominated	Weakly dominated
Cost of surgery = $42.6	111	236	Weakly dominated	Weakly dominated
Cost of surgery = $106.5	184	296	Weakly dominated	Weakly dominated
Cost of spectacles = $3.3	142	250	Weakly dominated	Weakly dominated
Cost of spectacles = $6.5	145	278	Weakly dominated	Weakly dominated
Patient costs low end of 95% CI	133	250	Weakly dominated	Weakly dominated
Patient costs upper end of 95% CI	154	275	Weakly dominated	Weakly dominated
Baseline, standard of care = 10% self present at hospitals	150	262	Dominated	Dominated
Baseline, standard of care = 20% self present at hospitals	165	262	Dominated	Dominated
Baseline, standard of care = 30% self present at hospitals	221	262	Dominated	Dominated

Section S7. Exploratory Analysis on the Relationship between Case finding and Treatment Initiation Cost and Scale

To explore whether case finding and treatment initiation costs increase or decrease with scale, we plot a graph of average case finding and treatment initiation costs against the number of people screened by provider. We separate URE and cataracts, and categorize strategies into vision centers (blue) and all other strategies (orange). The results are presented below with the y-axis removed to protect institutional privacy. For this exploratory analysis, the exact numbers are not as important as the general shape of the relationship. For both URE and cataracts, the evidence suggests a positive linear relationship between average case finding and treatment initiation costs and number of people screened for non-vision center strategies. In contrast, vision center costs are relatively insensitive to scale.

Figure S7.1: Relationship between case finding and treatment initiation costs for URE against number of people screened. Vision centers (blue) and other strategies (school eye screening, door-to-door screening and vision camps, orange)

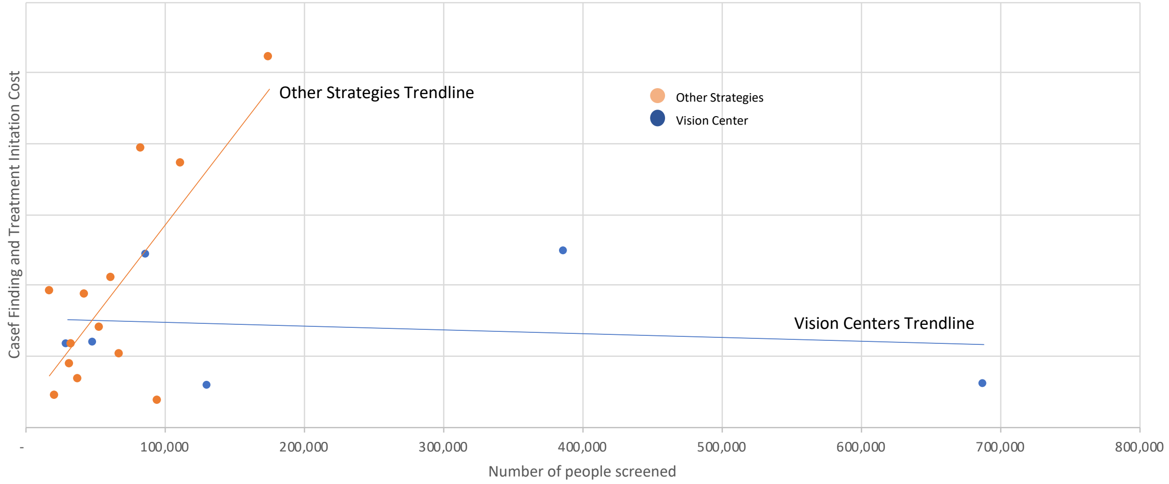

Figure S7.2: Relationship between case finding and treatment initiation costs for cataracts against number of people screened. Vision centers (blue) and other strategies (school eye screening, door-to-door screening and vision camps, orange).

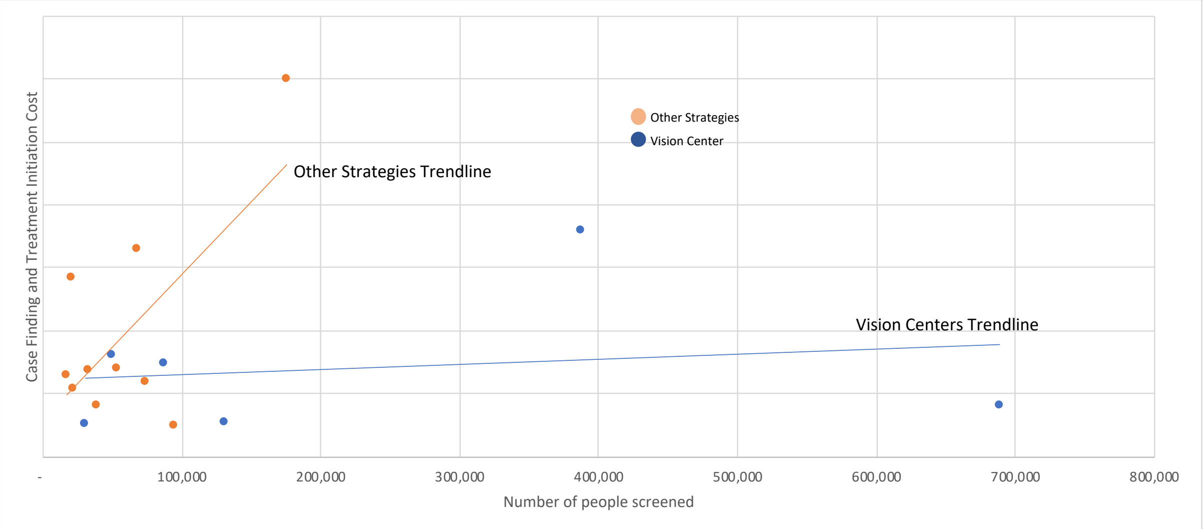

References for Supplementary Materials

1. Khanna RC, Sabherwal S, Sil A, et al. Primary eye care in India – The vision center model. *Indian J Ophthalmol*. 2020;68(2):333-339. doi:10.4103/ijo.IJO_118_19

2. Tang J, Liang Y, O'Neill C, Kee F, Jiang J, Congdon N. Cost-effectiveness and cost-utility of population-based glaucoma screening in China: a decision-analytic Markov model. *Lancet Glob Health*. 2019;7(7):e968-e978. doi:10.1016/S2214-109X(19)30201-3

3. Kovai V, Rao GN, Holden B, et al. An Estimate of Patient Costs and Benefits of the New Primary Eye Care Model Utilization Through Vision Centers in Andhra Pradesh, India. *Asia Pac J Public Health*. 2010;22(4):426-435. doi:10.1177/1010539510370779

4. Le HG, Ehrlich JR, Venkatesh R, et al. A Sustainable Model For Delivering High-Quality, Efficient Cataract Surgery In Southern India. *Health Aff (Millwood)*. 2016;35(10):1783-1790. doi:10.1377/hlthaff.2016.0562

5. IHME. *Global Burden of Disease 2019*.; 2021. https://vizhub.healthdata.org/gbd-compare/

6. Ministry of Health and Family Welfare. *National Blindness and Visual Impairment Survey India 2015-2019 - A Summary Report*.; 2020.

7. Ehrlich JR, Ramke J, Macleod D, et al. Association between vision impairment and mortality: a systematic review and meta-analysis. *Lancet Glob Health*. 2021;9(4):e418-e430. doi:10.1016/S2214-109X(20)30549-0

8. Wong B, Singh K, Kanna RK, et al. The Economic and Social Costs of Visual Impairment and Blindness in India. *Indian J Ophthalmol*. 2022;70(10):3470-3475. doi:10.4103/ijo.IJO_502_22
